# Supplementary material for: Estimate the burden of sexual dysfunction due to non-communicable diseases in Ethiopia: Systematic review and meta-analysis
Source: PLoS One. 2021 Oct 28;16(10):e0258938. doi: 10.1371/journal.pone.0258938 (PMC8553047; doi:10.1371/journal.pone.0258938)
Supplement: S1 Table — (DOCX) [file pone.0258938.s001.docx]

**JBI critical appraisal checklist for studies reporting prevalence data**

| **JBI Critical Appraisal Checklist** | Authors | | | | | |
| --- | --- | --- | --- | --- | --- | --- |
|  | Ejigu et al. (2019) | Asefa et al. (2019) | Fanta et al. (2018) | Gerensea H. et al. (2018) | Zewlde KH. et.al. (2017) | Tesfaye et al. (2020) |
| Was the sample frame appropriate to address the target population | Yes | Yes | Yes | Yes | Yes | Yes |
| Were study participants sampled in an appropriate way | Yes | Unclear | No | Yes | Yes | No |
| Was the sample size adequate | Yes | No | No | Yes | No | No |
| Were the study subjects and the setting described in detail? | Yes | Yes | Yes | Yes | Yes | Yes |
| Was the data analysis conducted with sufficient coverage of the identified sample? | Yes | Yes | Yes | Yes | Yes | Yes |
| Were valid methods used for the identification of the condition? | Unclear | Yes | Unclear | Unclear | Yes | Unclear |
| Was the condition measured in a standard, reliable way for all participants? | Yes | Yes | Yes | Yes | Yes | Yes |
| Was there appropriate statistical analysis? | Yes | Yes | Yes | Yes | Yes | Yes |
| Was the response rate adequate, and if not, was the low response rate managed appropriately? | Yes | Yes | Yes | Yes | Yes | Yes |
| Total score | 8/9*100=89% | 7/9*100=78% | 6/9*100=67% | 8/9*100=89% | 8/9*100=89% | 6/9*100=67% |
