# Supplementary material for: Estimate the burden of sexual dysfunction due to non-communicable diseases in Ethiopia: Systematic review and meta-analysis
Source: PLoS One. 2021 Oct 28;16(10):e0258938. doi: 10.1371/journal.pone.0258938 (PMC8553047; doi:10.1371/journal.pone.0258938)
Supplement: Attachment — Submitted filename: response to reviewer SD.pdf [file pone.0258938.s003.pdf]

Dear Academic Editor!

PLOS ONE

Response to Reviewers

I am happy to resubmit for publication version of "**Estimate the burden of sexual dysfunction due to non-communicable disease in Ethiopia: systematic review and meta-analysis**" for a review as original research in PLOS ONE.

The comments of the editor and the reviewers were highly insightful and enabled us to greatly improve the quality of our manuscript. Therefore, based on the editor's and the reviewers' concerns we have made extensive edition in our manuscript. Particularly, we have extensively edited the manuscript by a professional language editor (English-language instructors of Debre Berhan University thoroughly edited the manuscript for language usage, spelling, and grammar) before submitting the revised version. The formatting of the text and document (text sizes and grammatical errors) was also edited. We have addressed yours' concerns in a point by point format.

We look forward to hearing from you at your earliest convenience.

Thank you for your consideration of this manuscript!

Kind regards,

Akine Eshete

On behalf of authors

The authors of this study made a precious effort to investigate the burden of non-communicable diseases (NCDs) on sexual dysfunction in Ethiopia, especially focusing on the quality of life of patients with NCDs. This systematic review and meta-analysis tried to investigate the mentioned notion through a comprehensive review and statistical analyses. However, some comments are necessary to be addressed and I provide them below:

1. The title of the manuscript is not fully appropriate and consistent with the study aim. The last sentence of the background part of the abstract "estimate the burden of sexual dysfunction due to non-communicable disease in Ethiopia" is more appropriate for such investigation.
   Response: We thank you very much for this important recommendation. We edited the title accordingly.
2. "Non-communicable disease" is better to be replaced by "Non-communicable diseases" in the last sentence of the background part of the abstract.
   Response: thank you, for your important comment. Based on your recommendations, we have made revision of the manuscript accordingly

3. "Noncommunicable Diseases" is correct for keywords according to the MeSH database of PubMed.
   Response: We would like to say thank you once again for your fruitful comments. This has been addressed as your recommendation.
4. The first two sentences of the introduction, exploring the statistics, need to be explained which year the belong to, provided in the reference.
   Response: Based on your recommendations, we have addressed the issue. In line 44-48.
5. A grammatical and linguistic edit is essential for this manuscript, as the numerous issues are apparent in it and make understanding the provided draft difficult.
   Response: thank you very much for your important comment. We acknowledge that English is not our first language and we have edited the manuscript by an English language instructor. Based on your recommendations, we have made revision of the manuscript accordingly.
6. 2nd paragraph of introduction: are NCDs comorbidities of sexual dysfunction or sexual dysfunction is a comorbidity of NCDs?
   Response: Based on your recommendations, we have addressed the issue. In line 55-56.
7. 4th paragraph of introduction: references for the first sentence should be merged in a citation manager.
   Response: Based on your recommendations, we have addressed the issue. In line 67-69.

8. The knowledge gap is not well addressed in the introduction of the manuscript and needs to be explained more.
   Response: Based on your recommendations, we have addressed the issue. In line 49-54.
9. The "Objective" and "Research question" after the introduction, could be merged into the introduction part and is a repeat of the mentioned notions in the introduction.
   Response: Based on your recommendations, we have addressed the issue. In line 73-77.
10. In methods and materials: in inclusion criteria, types of included NCDs should be provided specifically.
    Response: Based on your recommendations, we have addressed the issue.
11. In methods and materials: in inclusion criteria, what is the reference for the standardized tool for sexual dysfunction measurement? Is it validated before for the Ethiopian population?
    Response: Based on your recommendations, we have addressed the issue. In line 95-102.

12. In methods and materials: in exclusion criteria, are there any criteria of exclusion for any of NCDs, participants, or sexual dysfunction disorders? The exclusion criteria could be more detailed based on the aims of this study.

    Response: Based on your recommendations, we have addressed the issue.

13. In methods and materials: why didn't authors search the Scopus database?

    Response: We thank you very much for this important recommendation and comment. It was writing problems, so it is corrected accordingly

14. In methods and materials: the search period is stated to be from inception to 2020 and also a period of 2000-2020? Please clarify which one is the searched period exactly.

    Response: We thank you very much. Included study published from 2000-2020
    Searching time was from May 1-31/ 2020 and updated June 5/2020

15. In methods and materials: there is not so much information about the screening process of the articles and about how did the screening was done by one or two authors.

    Response: Based on your recommendations, we have addressed the issue.
    In line 29-32.

16. The terms "gray literature" and "grey literature" are used both. Please, unify this term through the manuscript.

    Response: Based on your recommendations, we have addressed the issue.
    Gray literature is information produced by government agencies, academic institutions, and also the for-profit sector that is not typically made available by commercial publishers. Examples of **gray literature** include: Reports. Proceedings. Dissertations and theses.The term **grey literature** refers to **research** that is either unpublished or has been published in non-commercial form. Examples of **grey literature** include: government reports. policy statements and issues papers.

17. Results, Table 1, "type of measurement" column measures are not discussed in the Methods section and should be elaborated in detail and the issue of difference of measures among different studies should be addressed and discussed. How did the authors compare studies while the measure differed?

    Response: Based on your recommendations, we have addressed the issue.

18. Subgroup analysis and sensitivity analysis explanations should be provided in the methods section instead of the results section.

    Response: Based on your recommendations, we have addressed the issue.

19. "Fig" and "Figure" terms should be unified as "Figure" in the manuscript.

    Response: we did based on the comments

20. Why there is no Figure 4 in or at the end of the manuscript?

    Response: we did based on the comments

21. The terms "non-communicable patients" and "non-communicable clients" are not proper provided in many parts of the manuscript.

    Response: Based on your recommendations, we have addressed the issue.

22. In discussion: did authors find any explanation for the higher prevalence of sexual dysfunction in patients with mental disorders? Please include some information about this important finding in the discussion section.
    Response: Based on your recommendations, we have addressed the issue.
    In line 219-223
23. In discussion: findings are not discussed well enough, and it seems authors could use literature to highlight the importance of the investigated notion and compare results with other similar studies.
    Response: Based on your recommendations, we have addressed the issue.
    In line 219-223
24. Also, authors could replace the paragraph "Implications of the study findings" into the discussion as a part for policymakers and public health authorities, besides the provided implications for the clinicians.
    Response: Based on your recommendations, we have addressed the issue.
25. Figure 5 legend, "log" should be corrected in the legend and "LogitSD" on the figure.

    Response: Based on your recommendations, we have addressed the issue.

26. The six articles included in the final analysis and report is inconsistent in Table 1 and Supplementary Table 1 in comparison to Figures 2 and 3. "Gerensea H. et al. (2018)" and "Zewlde KH. et.al. (2017)" are in tables, but "Walle et al. (2018)" and "Seid et al. (2017)" in figures.
    Response: we did based on the comments
27. Included paper in analyses with reference numbers 15 *"tesfaye t, bayisa m, mesfin N: Prevalence and associated factors of erectile dysfunction among men DM patients in Gondar university hospital, Gondar Ethiopia. In.: Research Square; 2020"* and reference number 18 *"Gebrezgabhier G, Desta H, Berhe T, Hailu E, Gebrehiwot F, Kifle Y: Prevalence and associated factors of Female Sexual Dysfunction among Female Population in Aksum Town, Tigray Region, Ethiopia, 2019. A Community Based Cross Sectional Study. In.: Research Square; 2019"* included in reports are preprints in the Research Square database and not peer-reviewed articles. And this in contrast with your methods!
    Response: We thank you very much for this important recommendation. We consider both published and unpublise research work. We modify the method parts
28. Included paper in analyses with reference numbers 16 *"Gerensea H, Tarko S, Zenebe Y, Mezemir R, Walle B, Lebeta KR, Fita YD, Abdissa HG: Prevalence of erectile dysfunction and associated factors among diabetic men attending the diabetic clinic at Felege Hiwot Referral Hospital, Bahir Dar, North West Ethiopia, 2016. BMC endocrine disorders 2018, 11(1):130*" is not traceable in online databases!

Response: We thank you very much for this important recommendation. We correct like Bizuayehu Walle, Kidist Reba Lebet , Yamrot Debela Fitaand Hordofa Gutema Abdissa. *Prevalence of erectile dysfunction and associated factors among diabetic men attending the diabetic clinic at Felege Hiwot Referral Hospital, Bahir Dar, North West Ethiopia, 2016. BMC endocrine disorders 2018, 11(1):130*"

29. Included paper in analyses with reference numbers 17 "Zewlde KH, Muluneh NY, Seraj ZR, GebreLibanos MW, Bezabih YH, Seid A: Prevalence and determinants of erectile dysfunction among diabetic patients attending in hospitals of central and northwestern zone of Tigray, northern Ethiopia: a cross-sectional study. BMC neurology 2017, 17(1):16" is not traceable in online databases!

    Response: We thank you very much for this important recommendation. We correct like Awole Seid , Hadgu Gerensea, Shambel Tarko, Yosef Zenebe and Rahel Mezemir. Prevalence and determinants of erectile dysfunction among diabetic patients attending in hospitals of central and northwestern zone of Tigray, northern Ethiopia: a cross-sectional study. BMC neurology 2017.

30. Included paper in analyses with reference numbers 20 *"Ejigu AK: Sexual dysfunction and associated factors among patients with epilepsy at Amanuel Mental Specialty Hospital, Addis Ababa - Ethiopia. Turkish journal of obstetrics and gynecology 2019, 19(1):255"* is not published in the mentioned journal provided in the citation, instead of in the BMC Neurology journal!

Response: We thank you very much, we checked and published at BMC Neurology journal
