# Supplementary material for: Estimate the burden of sexual dysfunction due to non-communicable diseases in Ethiopia: Systematic review and meta-analysis
Source: PLoS One. 2021 Oct 28;16(10):e0258938. doi: 10.1371/journal.pone.0258938 (PMC8553047; doi:10.1371/journal.pone.0258938)
Supplement: Attachment — Submitted filename: Response to reviewre.docx [file pone.0258938.s004.docx]

**Dear Academic Editor!**

**PLOS ONE**

**Response to Reviewers**

I am happy to resubmit for publication version of “**Estimate the burden of sexual dysfunction due to non-communicable disease in Ethiopia: systematic review and meta-analysis**” for a review as original research in PLOS ONE.

The comments of the editor and the reviewers were highly insightful and enabled us to greatly improve the quality of our manuscript. Therefore, based on the editor’s and the reviewers’ concerns we have made extensive edition in our manuscript. Particularly, we have extensively edited the manuscript by a professional language editor (English-language instructors of Debre Berhan University thoroughly edited the manuscript for language usage, spelling, and grammar) before submitting the revised version. The formatting of the text and document (text sizes and grammatical errors) was also edited. We have addressed yours’ concerns in a point by point format.

We look forward to hearing from you at your earliest convenience.

Thank you for your consideration of this manuscript!

Kind regards,

**Akine Eshete**

On behalf of authors

The authors of this study made a precious effort to investigate the burden of non-communicable diseases (NCDs) on sexual dysfunction in Ethiopia, especially focusing on the quality of life of patients with NCDs. This systematic review and meta-analysis tried to investigate the mentioned notion through a comprehensive review and statistical analyses. However, some comments are necessary to be addressed and I provide them below:

Reviewer #1: Question 1
My comments in the previous review have been adequately taken care of, the objective of the study now agrees with the findings

Reviewer #2: Authors of this study revised the manuscript based on the provided comments. The changes were noticeable. However, some major points still remain to be answered and considered in the manuscript. A response to the authors’ letter is provided below, by number of previous comments of mine:

1. Comment well addressed. Title is edited.
2. Comment not addressed (“Non-communicable disease” is better to be replaced by “Non-communicable diseases” in the last sentence of the background part of the abstract.)

Response: We thank you very much for this important recommendation. We edited it accordingly.

3. Comment well addressed.
4. Comment well addressed.
5. Comment for language edit is addressed. However a final round of language edit is necessary prior to publication.

Response: thank you very much for your important comment. We acknowledge that English is not our first language and we have edited the manuscript by an English language instructor. Based on your recommendations, we have made revision of the manuscript accordingly

6. Comment well addressed.
7. Comment well addressed.
8. Comment not addressed. I meant the knowledge gap about the sexual dysfunction and NCDs in the literature. Authors added an irrelevant part about knowledge on diabetes to the introduction!

Response: thank you very much for your important comment, we deleted

9. Comment well addressed.
10. Comment well addressed.
11. Comment partially addressed. Please add the full terms of IIEF and CSFQ. Also, please add whether the tool is standardized for the Ethiopian population or not.

Response: thank you very much for your important comment; we adopted already standardized tools based on the context of Ethiopia

12. Comment well addressed.
13. Comment partially addressed. Please add the exact time period of search to the text (In methods and materials: the search period is stated to be from inception to 2020 and also a period of 2000-2020? Please clarify which one is the searched period exactly.)

Response: We thank you very much for this important recommendation. We edited on line 116 and 117

14. Comment not addressed. The screening process is different from the data extraction. This comment is a major one and still needs to be answered (In methods and materials: there is not so much information about the screening process of the articles and about how did the screening was done by one or two authors.)

Response: We thank you very much for this important recommendation. We edited on line 129 and 136

15. Comment not addressed. Please provide reference for the difference you mean between gray and grey literature.

Response: We thank you very much for this important recommendation. We edited on line 116

16. Comment partially addressed. Types of measurement are added to the methods. But still authors did not answer how they compare studies when the measurement tool is different. As the man concern, how did authors conduct the meta-analysis when the measurement tools were different among the included studies? It is recommended to do meta in each category separately.

Response: We thank you very much for this important recommendation; we understood that our outcome interest is sexual dysfunction. We summarized the prevalence of sexual dysfunction, which was assessed the same tool.

17. Comment not addressed (Subgroup analysis and sensitivity analysis explanations should be provided in the methods section instead of the results section).

Response: We thank you very much for this important recommendation. We edited on line from 207 to 209

18. Comment well addressed
19. Comment well addressed.
20. Comment well addressed.
21. Comment well addressed.
22. Comment well addressed
23. Comment well addressed
24. Comment not addressed (Figure 5 legend, “log” should be corrected in the legend and “LogitSD” on the figure.)

Response: We thank you very much for this important recommendation. We edited it accordingly.

25. Comment partially addressed. Reference to “Walle (2018)” is corrected. Reference to “Seid (2017)” is incorrect in the bibliography. Also, statistics of mild, moderate, and severe SD in Table 1 are not consistent with numbers in the original paper! Also, data for “Ejigu (2019)” has a problem as the total prevalence of SD was 63.9 and is stated to be 63.3.

Response: We thank you very much for this important recommendation. We edited it accordingly.

26. Comment well addressed.
27. Comment well addressed.
28. Comment partially addressed. Citation number 34 is still incorrect in the bibliography of manuscript. Please correct it as you answered the comment. It is corrected accordingly

Response: We thank you very much for this important recommendation. We edited it accordingly.

29. Comment not addressed. Please correct the citation in the bibliography f manuscript. . It is corrected accordingly

Response: We thank you very much for this important recommendation. We edited it accordingly.

Reviewer #3: The language used throughout the manuscript is sub-standard and needs significant improvement. Several of the paragraphs are constructed poorly, words are used incorrectly, poor grammar is used, some paragraphs include very long incomprehensible sentences. Acronyms are used in the manuscript sometimes without explanation, and at other times after an acronym is used, the full term is reused in the manuscript., instead of the acronym. The manuscript contains repetition. The Discussion is not cohesive or logical. Many loose statements are made with no references. The Conclusion of the manuscripts includes a repetition of the study results.

Response: We thank you very much for these important comments. We corrected it accordingly the comments
