# Supplementary material for: Estimate the burden of sexual dysfunction due to non-communicable diseases in Ethiopia: Systematic review and meta-analysis
Source: PLoS One. 2021 Oct 28;16(10):e0258938. doi: 10.1371/journal.pone.0258938 (PMC8553047; doi:10.1371/journal.pone.0258938)
Supplement: Attachment — Submitted filename: Response to Reviewers.docx [file pone.0258938.s005.docx]

**Dear Academic Editor!**

**PLOS ONE**

**Response to Reviewers**

I am happy to resubmit for publication version of “**Estimate the burden of sexual dysfunction due to non-communicable disease in Ethiopia: systematic review and meta-analysis**” for a review as original research in PLOS ONE.

The comments of the editor and the reviewers were highly insightful and enabled us to greatly improve the quality of our manuscript. We have addressed yours’ concerns in a point by point format.

Thank you for your consideration of this manuscript!

Kind regards,

**Akine Eshet**

**Comments**
Journal Requirements:

Please review your reference list to ensure that it is complete and correct. If you have cited papers that have been retracted, please include the rationale for doing so in the manuscript text, or remove these references and replace them with relevant current references. Any changes to the reference list should be mentioned in the rebuttal letter that accompanies your revised manuscript. If you need to cite a retracted article, indicate the article’s retracted status in the References list and also include a citation and full reference for the retraction notice.

Response: thank you, for your important comment. We check and all references are complete and correct in line with the journal requirement.
**Comments of reviewer**

1. Line 46 - The (WHO 2018) funding showed that 41 million cases and 57 million deaths - - Sentence unclear may be incomplete?
2. Line 47-49 - Sentences unclear - poor sentence construction and grammar
3. Lines 60-62 - long sentence and poor sentence construction
4. Lines 63-64 - incorrect grammar- “.....due to an “absent
5. The acronym SD is introduced in Line 63, but not used in the many other places following where sexual dysfunction is used (lines 68, 69, 70,73-74,78)
6. Lines 68-79 - unclear paragraph –

Response: thank you, for your important comment. Based on your recommendations, we have made revision accordingly (Line 46-79)

7. Lines 68-72 - is this about Ethiopia or worldwide (global)??

Response: thank you, for your important comment. In the paragraph we mean that patients with non-communicable diseases in Ethiopia, we corrected

8. Lines 72-75 - incorrect grammar-...., this systematic review and meta-analysis were aimed to synthesize.....
9. Lines 76-79 - poor sentence construction and grammar
10. Lines 89-91 - incomplete sentence
11. Lines 94-100- Paragraph includes poor sentence construction and grammar

Response: thank you, for your important comment. Based on your recommendations, we have made revision accordingly (line 72-100)

12. Lines 103-109 - unclear...Is that included as part of the Types of outcome measures?? Unclear what screening tool you are talking about.

Response: thank you, for your important comment. Types of outcome measures/ domain of sexual dysfunction/, finally after computing these parameterise, researchers categorize having sexual dysfunction and not.

13. Lines 118-120 - sentence incomplete
14. Line 124 - Acronym JBI used without explanation
15. Lines 124-127- Poor sentence construction
16. Line 129-135 - Poor grammar
17. Line 136- Unclear, poor sentence construction
18. Lines 141-150 - Poor paragraph/sentence construction, incomplete sentences, poor grammar
19. Lines 153-154 - incomplete sentence, incomprehensible
20. Lines 159 - 161 - poor sentence construction/grammar

Response: thank you, for your important comment. Based on your recommendations, we have made revision accordingly (line 118-161)

21. Lines 171-172 - About 198 articles were excluded, Could the exact number of articles excluded be provided

Response: thank you, for your important comment. Using the developed Boolean operators any one checks and confirms the available studies, we excluded by considering the title and reading of abstracts

22. Lines 175-176 - Poor grammar
23. Lines 195-197 - unclear paragraph
24. Lines 206-208 - To discover the potential reasons for the heterogeneity by subgroup meta-analyses were analyzed...awkward sentence/paragraph.

Response: thank you, for your important comment. Based on your recommendations, we have made revision accordingly (line 118-161)

**DISCUSSION**

While improvements have been made to the Discussion by including references, the grammar and sentence construction still needs to be improved.

25. Lines 235-236 - poor grammar
26. Lines 239 - “sexual dysfunctions” - poor grammar
27. Line 240 - “complications was critical”- wrong tense
28. Lines 246-248 - poor sentence construction
29. Lines 251-252 - poor sentence construction
30. Lines 252-253 - poor sentence construction
31. Lines 257-259 - unclear sentence
32. Lines 262-266 - Very long unclear sentence
33. Lines 266-267 - unclear sentence
34. Lines 269 - 271 - poor sentence construction
35. Lines 274-279 - Poor construction of sentences and paragraph
36. Lines 280-281 - unclear sentence

Response; Thanks this important comments, we did accordingly (line 235-281)

37. Lines 285 -286 - It is “mandatory” to include screening and management ..... - Mandatory is a very strong word. - Please include reference.

Response; Thanks this important comments, we cited

38. Lines 294-302 - Poor grammar, very long sentence and tense confusion in the paragraph

Conclusion

39. The conclusion remains weak with results repeated. Requires revision.

Response; Thanks this important comments, we did accordingly
