# Supplementary material for: Estimate the burden of sexual dysfunction due to non-communicable diseases in Ethiopia: Systematic review and meta-analysis
Source: PLoS One. 2021 Oct 28;16(10):e0258938. doi: 10.1371/journal.pone.0258938 (PMC8553047; doi:10.1371/journal.pone.0258938)
Supplement: Attachment — Submitted filename: Response to reviewers.docx [file pone.0258938.s006.docx]

**Dear Academic Editor!**

**PLOS ONE**

**Response to Reviewers**

I am pleased to resubmit the revised version of "Estimate the burden of sexual dysfunction related to non-communicable disease in Ethiopia: systematic review and meta-analysis" to PLOS ONE for publication as original research.

The editor's and reviewers' comments were quite helpful, allowing us to significantly increase the quality of our article. As a result, we have made considerable revisions to our paper in accordance to the editor's and reviewers' comments.

Before submitting the revised version, the manuscript extensively edited by a professional language editor (English-language instructors of Debre Berhan University thoroughly edited the manuscript for language usage, spelling, and grammar). The text and document's formatting (text sizes and grammatical errors) were also edited. Your concerns have been addressed in a point-by-point approach.

We look forward to hearing from you at your earliest convenience.

Thank you for your consideration of this manuscript!

Kind regards,

**Akine Eshete**

On behalf of authors

The authors of this study made a precious effort to investigate the burden of non-communicable diseases (NCDs) on sexual dysfunction in Ethiopia, especially focusing on the quality of life of patients with NCDs. This systematic review and meta-analysis tried to investigate the mentioned notion through a comprehensive review and statistical analyses. However, some comments are necessary to be addressed and I provide them below:

Reviewer #4

Besides identification of abbreviations for their first appearance (e.g., IIEF-5)

Response: We thank you very much for this important recommendation. We edited it accordingly on line 91

Table 1 needs abbreviations and explanations independent of main txt and all should be mentioned at footnote (e.g., SNNPR, ED...).

Response: We thank you very much for this important recommendation. We edited it accordingly on line 170

I believe the first sentence of line 190 is not complete or it is just vague.

Response: We thank you very much for this important recommendation. We edited it accordingly on line 182.

The PRISMA chart is excruciatingly messy and inconsistent. Please revise this part and unify numbers with the text.

Response: We thank you very much for this important recommendation. We tried to modify based on the comments.

Reviewer #5

1. Line 48, “of from” is not grammatically correct.

Response: We thank you very much for this important recommendation. We edited it accordingly from line 47-48.

2. Line 49 “It” must be substituted with "NCDs".

Response: We thank you very much for this important recommendation. We edited it accordingly

3. Line 54, “Hypertension” should not be capitalized.

Response: We thank you very much for this important recommendation. We edited it accordingly on line 54
Methods:
1. The conducted search strategy is up to June 2020. This strategy should be updated.

Response: We thank you very much for this important recommendation. We edited it accordingly
2. In Figure 1, in the screening section, the authors have identified that 198 studies have been excluded. The exclusion might be due to some reasons including “review article”, “not relating to the subject” and etc. So, these reason with the numbers of articles in each exclusion section should be written in this fellow chart.

Response: We thank you very much for this important recommendation. We edited it accordingly from line 157-158
Discussion:
1. The authors should add additional paragraphs regarding the solutions for preventing or screening sexual dysfunction.

Response: We thank you very much for this important recommendation. We edited it accordingly from line 285-310
2. The authors have not discussed the reasons and potential solutions for sexual dysfunction in patients with diabetes. This part should be included.

Response: We thank you very much for this important recommendation. We edited it accordingly from line 285-310
