# Supplementary material for: Estimate the burden of sexual dysfunction due to non-communicable diseases in Ethiopia: Systematic review and meta-analysis
Source: PLoS One. 2021 Oct 28;16(10):e0258938. doi: 10.1371/journal.pone.0258938 (PMC8553047; doi:10.1371/journal.pone.0258938)
Supplement: Attachment — Submitted filename: Reviewer comments.docx [file pone.0258938.s002.docx]

The authors of this study made a precious effort to investigate the burden of non-communicable diseases (NCDs) on sexual dysfunction in Ethiopia, especially focusing on the quality of life of patients with NCDs. This systematic review and meta-analysis tried to investigate the mentioned notion through a comprehensive review and statistical analyses. However, some comments are necessary to be addressed and I provide them below:

1. The title of the manuscript is not fully appropriate and consistent with the study aim. The last sentence of the background part of the abstract “estimate the burden of sexual dysfunction due to non-communicable disease in Ethiopia” is more appropriate for such investigation.
2. “Non-communicable disease” is better to be replaced by “Non-communicable diseases” in the last sentence of the background part of the abstract.
3. “Noncommunicable Diseases” is correct for keywords according to the MeSH database of PubMed.
4. The first two sentences of the introduction, exploring the statistics, need to be explained which year the belong to, provided in the reference.
5. A grammatical and linguistic edit is essential for this manuscript, as the numerous issues are apparent in it and make understanding the provided draft difficult.
6. 2^nd^ paragraph of introduction: are NCDs comorbidities of sexual dysfunction or sexual dysfunction is a comorbidity of NCDs?
7. 4^th^ paragraph of introduction: references for the first sentence should be merged in a citation manager.
8. The knowledge gap is not well addressed in the introduction of the manuscript and needs to be explained more.
9. The “Objective” and “Research question” after the introduction, could be merged into the introduction part and is a repeat of the mentioned notions in the introduction.
10. In methods and materials: in inclusion criteria, types of included NCDs should be provided specifically.
11. In methods and materials: in inclusion criteria, what is the reference for the standardized tool for sexual dysfunction measurement? Is it validated before for the Ethiopian population?
12. In methods and materials: in exclusion criteria, are there any criteria of exclusion for any of NCDs, participants, or sexual dysfunction disorders? The exclusion criteria could be more detailed based on the aims of this study.
13. In methods and materials: why didn’t authors search the Scopus database?
14. In methods and materials: the search period is stated to be from inception to 2020 and also a period of 2000-2020? Please clarify which one is the searched period exactly.
15. In methods and materials: there is not so much information about the screening process of the articles and about how did the screening was done by one or two authors.
16. The terms “gray literature” and “grey literature” are used both. Please, unify this term through the manuscript.
17. Results, Table 1, “type of measurement” column measures are not discussed in the Methods section and should be elaborated in detail and the issue of difference of measures among different studies should be addressed and discussed. How did the authors compare studies while the measure differed?
18. Subgroup analysis and sensitivity analysis explanations should be provided in the methods section instead of the results section.
19. “Fig” and “Figure” terms should be unified as “Figure” in the manuscript.
20. Why there is no Figure 4 in or at the end of the manuscript?
21. The terms “non-communicable patients” and “non-communicable clients” are not proper provided in many parts of the manuscript.
22. In discussion: did authors find any explanation for the higher prevalence of sexual dysfunction in patients with mental disorders? Please include some information about this important finding in the discussion section.
23. In discussion: findings are not discussed well enough, and it seems authors could use literature to highlight the importance of the investigated notion and compare results with other similar studies.
24. Also, authors could replace the paragraph “Implications of the study findings” into the discussion as a part for policymakers and public health authorities, besides the provided implications for the clinicians.
25. Figure 5 legend, “log” should be corrected in the legend and “LogitSD” on the figure.
26. The six articles included in the final analysis and report is inconsistent in Table 1 and Supplementary Table 1 in comparison to Figures 2 and 3. “Gerensea H. et al. (2018)” and “Zewlde KH. et.al. (2017)” are in tables, but “Walle et al. (2018)” and “Seid et al. (2017)” in figures.
27. Included paper in analyses with reference numbers 15 *“tesfaye t, bayisa m, mesfin N: Prevalence and associated factors of erectile dysfunction among men DM patients in Gondar university hospital, Gondar Ethiopia. In.: Research Square; 2020”* and reference number 18 *“Gebrezgabhier G, Desta H, Berhe T, Hailu E, Gebrehiwot F, Kifle Y: Prevalence and associated factors of Female Sexual Dysfunction among Female Population in Aksum Town, Tigray Region, Ethiopia, 2019. A Community Based Cross Sectional Study. In.: Research Square; 2019”* included in reports are preprints in the Research Square database and not peer-reviewed articles. And this in contrast with your methods!
28. Included paper in analyses with reference numbers 16 *“Gerensea H, Tarko S, Zenebe Y, Mezemir R, Walle B, Lebeta KR, Fita YD, Abdissa HG: Prevalence of erectile dysfunction and associated factors among diabetic men attending the diabetic clinic at Felege Hiwot Referral Hospital, Bahir Dar, North West Ethiopia, 2016. BMC endocrine disorders 2018, 11(1):130*” is not traceable in online databases!
29. Included paper in analyses with reference numbers 17 “Zewlde KH, Muluneh NY, Seraj ZR, GebreLibanos MW, Bezabih YH, Seid A: Prevalence and determinants of erectile dysfunction among diabetic patients attending in hospitals of central and northwestern zone of Tigray, northern Ethiopia: a cross-sectional study. BMC neurology 2017, 17(1):16” is not traceable in online databases!
30. Included paper in analyses with reference numbers 20 *“Ejigu AK: Sexual dysfunction and associated factors among patients with epilepsy at Amanuel Mental Specialty Hospital, Addis Ababa - Ethiopia. Turkish journal of obstetrics and gynecology 2019, 19(1):255”* is not published in the mentioned journal provided in the citation, instead of in the BMC Neurology journal!
